# Mechanism of Cerebralcare Granule® for Improving Cognitive Function in Resting-State Brain Functional Networks of Sub-healthy Subjects

**DOI:** 10.3389/fnins.2017.00410

**Published:** 2017-07-14

**Authors:** Jing Li, Hao Guo, Ling Ge, Long Cheng, Junjie Wang, Hong Li, Kerang Zhang, Jie Xiang, Junjie Chen, Hui Zhang, Yong Xu

**Affiliations:** ^1^Department of Humanities and Social Science, Shanxi Medical University Taiyuan, China; ^2^Department of Computer Science and Technology, Taiyuan University of Technology Taiyuan, China; ^3^Department of Medical Psychology, Shanxi Medical College for Continuing Education Taiyuan, China; ^4^Department of Psychiatry, First Hospital, First Clinical Medical College of Shanxi Medical University Taiyuan, China; ^5^Department of Radiology, First Hospital of Shanxi Medical University Taiyuan, China; ^6^MDT Center for Cognitive Impairment and Sleep Disorders, First Hospital, First Clinical Medical College of Shanxi Medical University Taiyuan, China

**Keywords:** cerebralcare granule®, cognitive function, functional magnetic resonance imaging, conventional network metrics, frequent subgraph mining, sub-health

## Abstract

Cerebralcare Granule® (CG), a Chinese herbal medicine, has been used to ameliorate cognitive impairment induced by ischemia or mental disorders. The ability of CG to improve health status and cognitive function has drawn researchers' attention, but the relevant brain circuits that underlie the ameliorative effects of CG remain unclear. The present study aimed to explore the underlying neurobiological mechanisms of CG in ameliorating cognitive function in sub-healthy subjects using resting-state functional magnetic resonance imaging (fMRI). Thirty sub-healthy participants were instructed to take one 2.5-g package of CG three times a day for 3 months. Clinical cognitive functions were assessed with the Chinese Revised Wechsler Adult Intelligence Scale (WAIS-RC) and Wechsler Memory Scale (WMS), and fMRI scans were performed at baseline and the end of intervention. Functional brain network data were analyzed by conventional network metrics (CNM) and frequent subgraph mining (FSM). Then 21 other sub-healthy participants were enrolled as a blank control group of cognitive functional. We found that administrating CG can improve the full scale of intelligence quotient (FIQ) and Memory Quotient (MQ) scores. At the same time, following CG treatment, in CG group, the topological properties of functional brain networks were altered in various frontal, temporal, occipital cortex regions, and several subcortical brain regions, including essential components of the executive attention network, the salience network, and the sensory-motor network. The nodes involved in the FSM results were largely consistent with the CNM findings, and the changes in nodal metrics correlated with improved cognitive function. These findings indicate that CG can improve sub-healthy subjects' cognitive function through altering brain functional networks. These results provide a foundation for future studies of the potential physiological mechanism of CG.

## Introduction

Sub-healthy status, a transitional stage between health and illness, refers to functional somatic syndromes or symptoms that are medically undiagnosed (Wu et al., 2016; Yang et al., 2016). Set by medical experts at the 2001 China Academic Symposium on Sub-health held (Jiang, 2006), the evaluation criteria for sub-healthy status are one or more of the following symptoms lasting 1 month over a year: (1) physically manifests itself in lassitude, limb numbness, dizziness, tinnitus, or headache; (2) psychological complaints of distraction, hypomnesis, dreaminess, insomnia, anxiety, or irritation; (3) a lack of vitality and interest; (4) decreased work capacity and self-perceived uncomfortable relationships with colleagues; (5) a weakened immune system; (6) seeing a doctor due to subjective discomfort symptoms without organic diseases. As a whole, sub-health can be characterized by a decline in physical function, mental function and social capacity (Bi et al., 2014). At the same time, sub-healthy status has attracted attention with regard to its significant negative effects such as insomnia (Wu et al., 2016). Sub-healthy status can also lead to cognitive difficulty including distraction and hypomnesis (Jiang, 2006). A research has suggested traditional Chinese medicine, a combination of plants, is more efficient to enhance cognitive function (Howes and Houghton, 2003). For instance, clausenamide isolated from a traditional Chinese herbal medicine has been used to enhance cognition (Chu et al., 2016). Cerebralcare Granule® (CG, Yangxue Qingnao Granule; Tasly Pharmaceutical Co., Ltd., Tianjin, China) is a Chinese herbal medicine compound mainly composed of *Angelica sinensis, Ligusticum chuanxiong*, Paeonia lactiflora Pall, Uncaria sinensis, Spatholobus suberectus, *Prunella vulgaris*, Hyriopsis cumingii, *Rehmannia glutinosa*, Cassia tora, *Corydalis yanhusuo*, and Asarum sieboldii Miquel. It was approved by the China State Food and Drug Administration in 1996 for treating headaches, insomnia, and vertigo (Sun et al., 2010; Wu C. H. et al., 2013). Some researchers, including our group (Xu et al., 2015; Guo et al., 2016), have begun to focus on how CG improves sub-healthy status (Tong, 2006) and cognitive function (Qu et al., 2016; Zhao et al., 2017).

Animal and clinical evidence collected over the past decade suggests that CG can improve cognitive function. Researchers demonstrated that chronic CG treatment could attenuate D-gal-induced memory impairment in rats performing the Morris water maze test (Qu et al., 2016). Our group has used molecular biotechnology to investigate the mechanism of CG, and the results suggested that CG can attenuate cognitive impairment in rats continuously overexpressing microRNA-30e (Xu et al., 2015). On the other hand, a clinical study of post-stroke patients showed that CG use led to greater cognitive improvement than aspirin (Zhao et al., 2017). Other groups demonstrated that ischemia-induced cognitive impairment could be ameliorated by CG (Xu et al., 2009; Sun et al., 2010; Zhang X. X. et al., 2016). In addition, CG has been widely used to treat chronic cerebral circulation insufficiency (Xiong et al., 2011; Wu C. H. et al., 2013), which is often associated with cognitive impairment (Hirai, 1991).

Functional magnetic resonance imaging (fMRI) is a non-invasive technique that can map brain function (Gore, 2003) and has been utilized to evaluate cognitive function. Some researchers have summarized current trends of fMRI to evaluate cognitive function (Bigler, 2014). Notably, improvement of cognitive function was associated with specific brain connective alterations (Burgess et al., 2010; Wang et al., 2011). A growing number of studies inferred that different brain functional networks indicate various cognitive domains such as intelligence (Simos et al., 2013; Wu K. et al., 2013). fMRI has previously been used to investigate biological mechanisms underlying traditional Chinese medicines such as Bushen capsules (Zhang et al., 2015; Zhang J. Y. et al., 2016) and Congrongyizhi capsules (Zhang et al., 2014). Brain networks analysis, which can provide a global view on the whole-brain scale and capture the topological structure of brain's functional connectivity networks (Bullmore and Sporns, 2009; Rubinov and Sporns, 2010), was developed to improve fMRI data analysis. There are currently several connectivity approaches including seed-based connectivity analysis (Biswal et al., 1995), regional homogeneity (Zang et al., 2004), and independent component analysis (Beckmann et al., 2005). Although, these methodologies have been successfully applied to map brain connectivity networks from different perspectives, none can capture the topological brain network structure. In contrast, brain networks analysis approaches allow us to simultaneously map the functional connectivity patterns among all brain units and explore how the layout is organized and modulated in response to an intervention (Liu et al., 2012). The most common brain network method is conventional network metrics (CNM), which can be analyzed using metrics from modern network theory where a network is considered as a set of nodes and edges (Sporns and Kötter, 2004; Butts, 2009). Herein, nodes represent brain regions, and edges represent the statistical correlation in fMRI signals across different regions. CNM is a powerful tool for identifying group differences in the connectivity strength of clusters of interconnected nodes (Harrington et al., 2015). The innovative method of frequent subgraph mining (FSM) technique has also attracted attention from neuroimaging researchers. FSM is an effective technique for the development of association rules, frequent items, sequential patterns, and tree mining (Wang et al., 2004) that is used in finding out the subgraph in common among a specific atlas. The core of the FSM lies in the subgraph isomorphism testing (Huan et al., 2003). A large number of methods have been proposed for FSM; because of graph traversal efficiency and subgraph mining efficiency, the gSpan algorithm (Yan and Han, 2002) has been widely used in many research fields including neuroimaging (Fei et al., 2014; Wang et al., 2014).

Based on preliminary clinical evidence that CG can improve cognitive function in sub-healthy people, we further explored the neural mechanism. We employed the Chinese Revised Wechsler Adult Intelligence Scale (WAIS-RC; Gong, 1982) and Wechsler Memory Scale (WMS; Gong et al., 1989) to assess cognition. We used two analytical approaches to process fMRI data to explore the underlying neurobiological mechanisms of CG. These experiments were designed to test the hypothesis that CG administration would improve the cognitive function of sub-healthy subjects by affecting brain functional networks.

## Materials and methods

### Participants

A total of 30 right-handed participants (six male; age range: 20–50 years, mean age: 34.50 ± 10.59 years; 66.67% of those with a bachelor or higher degree) were enrolled in CG group, and other 21 participants (five male; age range: 20–55 years, mean age: 31.95 ± 11.60 years; 66.67% of those with a bachelor or higher degree.) in control group. All participants were enrolled from Shanxi medical university or First Hospital of Shanxi Medical University by an open recruit, and on the principle of convenience sampling, 21 participants in the control group were from our another ongoing study. And considering their self-statements and professionals' diagnosis, they were determined under sub-healthy state according to the evaluation criteria for sub-healthy state (Jiang, 2006) by two independent attending physicians. No participant had a history of psychiatric disorder or neurological illness. None had metal implants or claustrophobia and were able to undergo MRI scans. The Ethics Committee of the Shanxi Medical University (Shanxi, China) approved this study, and written informed consent as approved by the local ethics committee was obtained from all subjects.

### Study design

In CG group, all participants completed resting state fMRI scans at baseline. The WAIS-RC and WMS were used to provide standard measures of participants' intellectual ability and memory ability, respectively. After the pre-test, subjects were instructed to take one 2.5-g package of CG three times a day for 3 months. WAIS-RC and WMS post-tests were acquired for all participants, and 29 underwent follow-up fMRI scans. Moreover, in control group, all participants completed WAIS-RC and WMS twice, 3 months apart.

### Image acquisition and preprocessing

A cohort of imaging data was acquired using a 3-T whole-body scanner (Magnetom Trio, Siemens Healthcare, Germany) located at Shanxi Provincial People's Hospital, Shanxi, China. A standard eight-channel phase-array head coil was used. Foam padding and headphones were used to limit head motion and reduce scanner noise. The subjects were instructed to rest with their eyes closed, not to think of anything in particular, and not to fall asleep. Functional images were collected transversely by using an echo-planar imaging sequence with the following settings: repetition time (TR) = 2,500 ms, echo time (TE) = 40 ms, field of view (FOV) = 24 × 24 cm^2^, image matrix size = 64 × 64, voxel size = 3 × 3 × 3 mm^3^, 32 transverse slices without slice gap, flip angle = 90°, and a total of 212 volumes for each subject.

Functional data preprocessing was carried out using the SPM8 toolbox (http://www.fil.ion.ucl.ac.uk/spm). The first 10 volumes of functional images were discarded due to the instability of the initial MRI signal and adaptation of participants to the circumstance. The remaining 202 images were first corrected for acquisition delay between slices and head motion. Three datasets were excluded from further analysis according to the criteria that translational parameters exceeded ±2.5 mm. The resulting images were normalized to the standard SPM8 echo-planar imaging template and resampled to 3-mm cubic voxels. The resultant normalized functional data underwent spatial smoothing (4-mm full width at half maximum [FWHM] Gaussian kernel) and removal of linear trends. Finally, cerebrospinal fluid (CSF), white matter and six head motion parameters were regressed.

### Network construction and CNM

To define the brain nodes, anatomical parcellation was performed using the automated anatomical labeling (AAL) template, segmenting the images into 90 anatomical regions of interests (ROIs; 45 ROIs for each hemisphere). To define the network edges, we calculated region-wise Pearson correlation coefficients and generated a 90 × 90 correlation matrix for each imaging dataset. Sparsity (S) was used to set the threshold, which was defined as the ratio of the number of real existing edges divided by the number of maximum possible edges in the network. In this study, threshold space was generated as Sϵ (5%, 40%). All of the network analysis was within this threshold space, and the interval was set to 1% throughout the study. For each selected threshold, we analyzed three nodal metrics including the degree (ki), betweenness centrality (bi), and nodal efficiency (ei) (the detailed mathematical definitions and interpretations of these network metrics are shown in Supplementary Material). To identify the hub regions, we calculated the degree of each node by averaging across all subjects in each group. A node with a high degree was considered a hub and was crucial to efficient network communication. More specifically, a brain region was defined as a hub when its nodal degree was at least 1 standard deviation higher than the average of the corresponding measure over the entire network.

### FSM

The gSpan algorithm first constructs a new lexicographic order among graphs and maps each graph into a unique minimum depth-first search (DFS) code as its canonical label. Based on the lexicographic order, gSpan utilizes the DFS strategy to efficiently mine frequent connected subgraph patterns. Finally, all subgraphs with non-minimal DFS code are pruned to avoid redundant candidate generations (the details of the algorithm description are shown in Supplementary Material).

### Statistical analysis

The differences of scaled scores between the CG group and the control group in pre-test were analyzed with independent-samples *t*-test using SPSS 17.0 software (SPSS Inc., Chicago, IL, USA). Before that, we had confirmed all scaled scores were normally distributed and had constant variances. Statistical significance of cognitive level was tested by two-way analysis of variance (ANOVA) with Treatment (CG, control) and Time (pre, post) factors using SPSS 17.0 software. And the significance level was defined as *p* < 0.05.

A non-parametric permutation test was conducted for 270 metrics (90 nodes, 3 metrics per node) to determine whether there were significant between-group differences, and the results were ranked according to *p*-value. Multiple linear regression was performed to remove the influence of sex and age [independent variables: area under the curve (AUC) of metric, dependent variable: age and sex]. Then, to correct for multiple comparisons, false discovery rate (FDR; Benjamini and Hochberg, 1995) was applied. Here, we used the code developed by David M. Groppe in 2010 on MATLAB platform (similarly hereinafter). And the significance level was defined as adjusted *p* < 0.05.

Furthermore, we used the Pearson correlation coefficient to assess correlations between nodal metric changes and cognitive function improvement using SPSS 17.0 software. To correct for multiple comparisons, FDR was applied. Significant correlation and marginal correlation were defined as adjusted *p* < 0.05 and adjusted *p* < 0.1, respectively.

## Results

### Behavioral data

**Figure 1:**
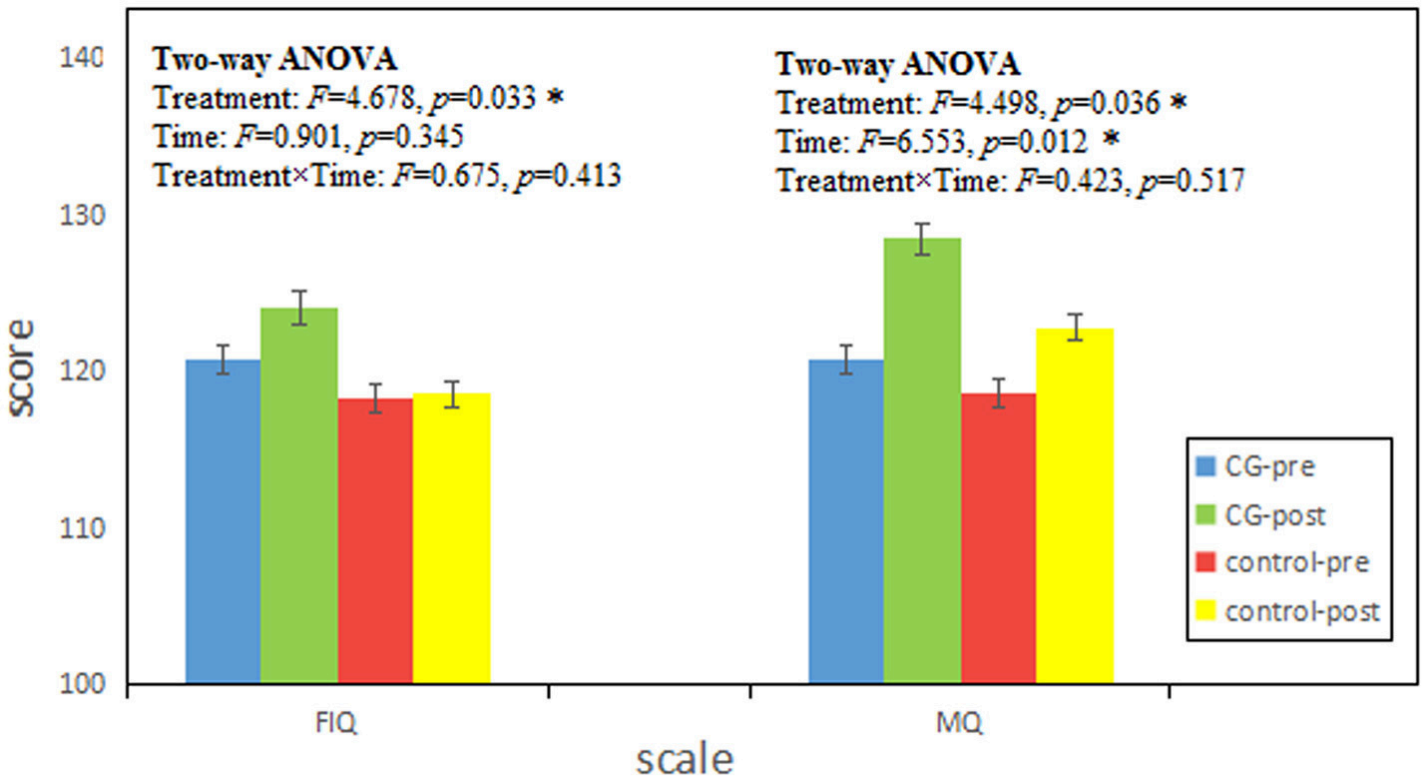
The results were tested by two-way ANOVAs with Treatment and Time. FIQ and MQ represent the full scales of Chinese Revised Wechsler Adult Intelligence Scale (WAIS) and Wechsler Memory Scale (WMS) respectively. Score is given as mean ± standard deviation. CG-pre represents the data of CG group in pre-test; CG-post represents the data of CG group in post-test; control-pre represents the data of control group in pre-test; control-post represents the data of control group in post-test. Treatment and Time represent the main effect of treatment (CG, control) and time (pre, post) respectively. Treatment × Time represents the interaction effect between treatment and time. *F* score represents the statistical value of Two-way ANOVAs. *p* represents the significance level (^*^p < 0.05).

Sex had no influence on intelligence and memory (the details of statistical results are shown in Supplementary Material), so we ignored sex differences in our participants. Independent-Samples *t*-test results of WAIS-RC and WMS in pre-test are shown in Table 1. There was no pre-test difference between the CG and control group (*p* > 0.05). And the results of two-way ANOVAs with Treatment and Time are shown in Figure 1. There was no significant interaction effect between treatment and time (Treatment × Time: *p* > 0.05), but the main effects of Treatment in FIQ (*p* = 0.033) and MQ (*p* = 0.036) were significant.

**Table 1:** Independent-Samples *t*-test results of WAIS-RC and WMS in pre-test.

	**Scale**	**Group**	***n***	**Means ± standard deviation**	**Leven's test for equality of variances**		***t*-test for equality of means**	
					***F***	***p_1_***	***T***	***p_2_***
1	FIQ	CG	30	120.833 ± 9.021	0.023	0.879	0.978	0.333
		Control	21	118.333 ± 8.924				
2	MQ	CG	30	120.367 ± 9.967	2.047	0.159	0.520	0.605
		Control	20	118.600 ± 14.080				

*FIQ and MQ represent the full scales of Chinese Revised Wechsler Adult Intelligence Scale (WAIS) and Wechsler Memory Scale (WMS), respectively. CG and control represent the CG group and the control group respectively. n represents the number of participants (in order to keep constant variance in MQ scores, one in the control group was rejected). Leven's test for equality of variances represents the results of homogeneity test of variance (“p_1_ > 0.05” represent two sets of data have constant variances). t-score represents the statistical value of Independent-Samples t-test. p_2_ represents the significance level. ES represents the effect size of the test*.

### CNM

**Figure 2:**
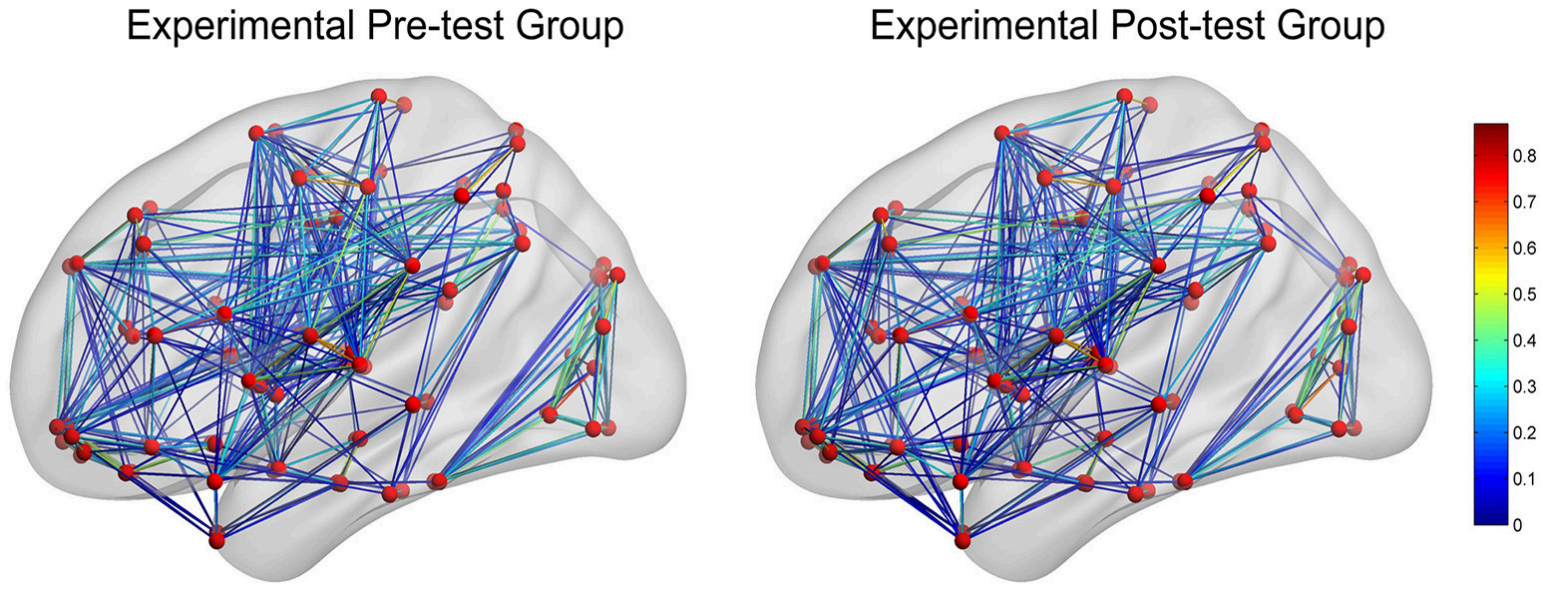
The networks construction of experimental pre-test group (baseline characteristics) and experimental post-test group (intervention outcomes). The red dots reflect nodes. The links reflect connections between nodes, and the color of links reflects connection strength.

**Figure 3:**
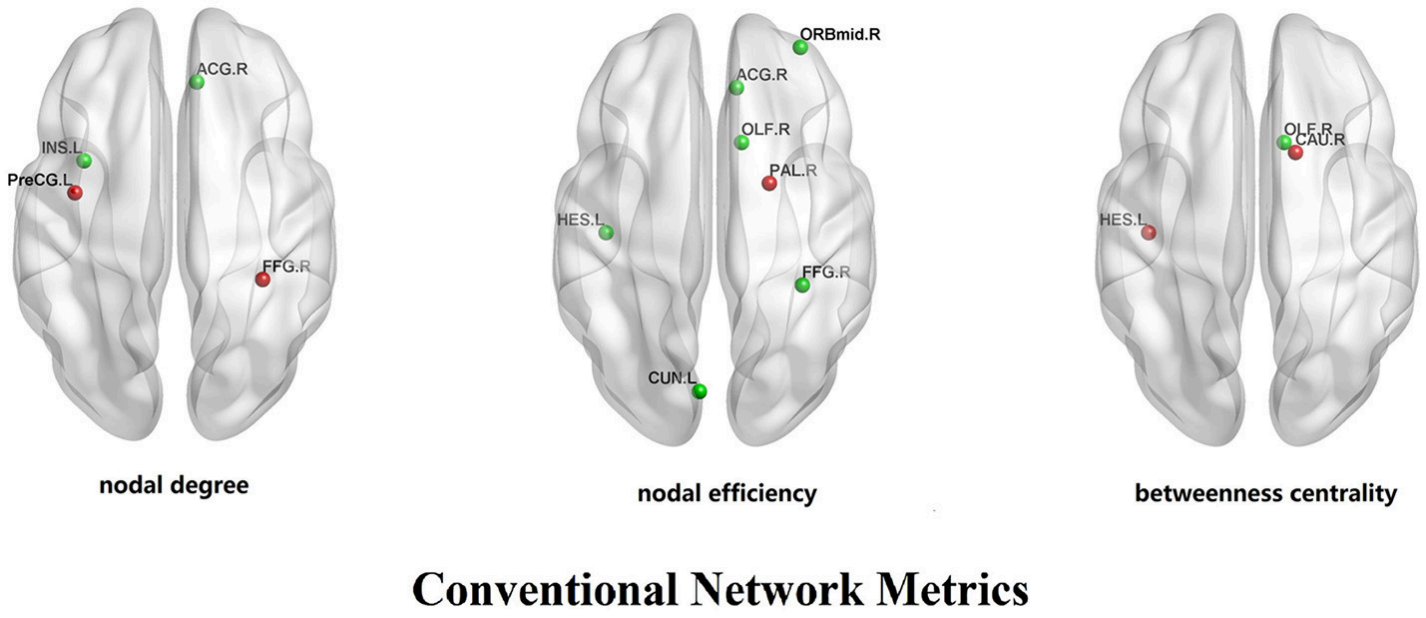
The differences of nodal characteristics (nodal degree, nodal efficiency, betweenness centrality) between experimental pre-test group and experimental post-test group. The red dots reflect the nodes whose nodal characteristics in pre-test group is more than post-test group. The green dots reflect the nodes whose nodal characteristics in post-test group is more than pre-test group.

In CG group, the network constructions are shown in Figure 2. Comparing the CNMs of baseline characteristics and intervention outcomes, participants exhibited significant differences in nodal graph metrics after taking CG. Specifically, we observed increased nodal degrees in the left insula (INS.L; *p* = 0.036) and right anterior cingulate and paracingulate gyri (ACG.R; *p* = 0.006), and decreased nodal degree in the left precentral gyrus (PreCG.L; *p* = 0.036) and right fusiform gyrus (FFG.R; *p* = 0.016); increased nodal efficiency was noted in the right middle frontal gyrus in orbital part (ORBmid.R; *p* = 0.036), right olfactory cortex (OLF.R; *p* = 0.036), ACG.R (*p* = 0.002), left cuneus (CUN.L; *p* = 0.036), FFG.R (*p* = 0.016) and left heschl gyrus (HES.L; *p* = 0.016) and decreased nodal efficiency in the right pallidum (PAL.R; *p* = 0.036) We also observed increased betweenness centrality in the OLF.R (*p* = 0.036), and decreased betweenness centrality in the right caudate nucleus (CAU.R; *p* = 0.016) and HES.L (*p* = 0.036). The differences of nodal characteristics between baseline and follow-up are shown in Figure 3. In addition, we found that the network hubs of baseline characteristics were the left rolandic operculum (ROL.L) and bilateral insula (INS.), while the network hubs after intervention were the right rolandic operculum (ROL.R) and INS.

### FSM

**Figure 4:**
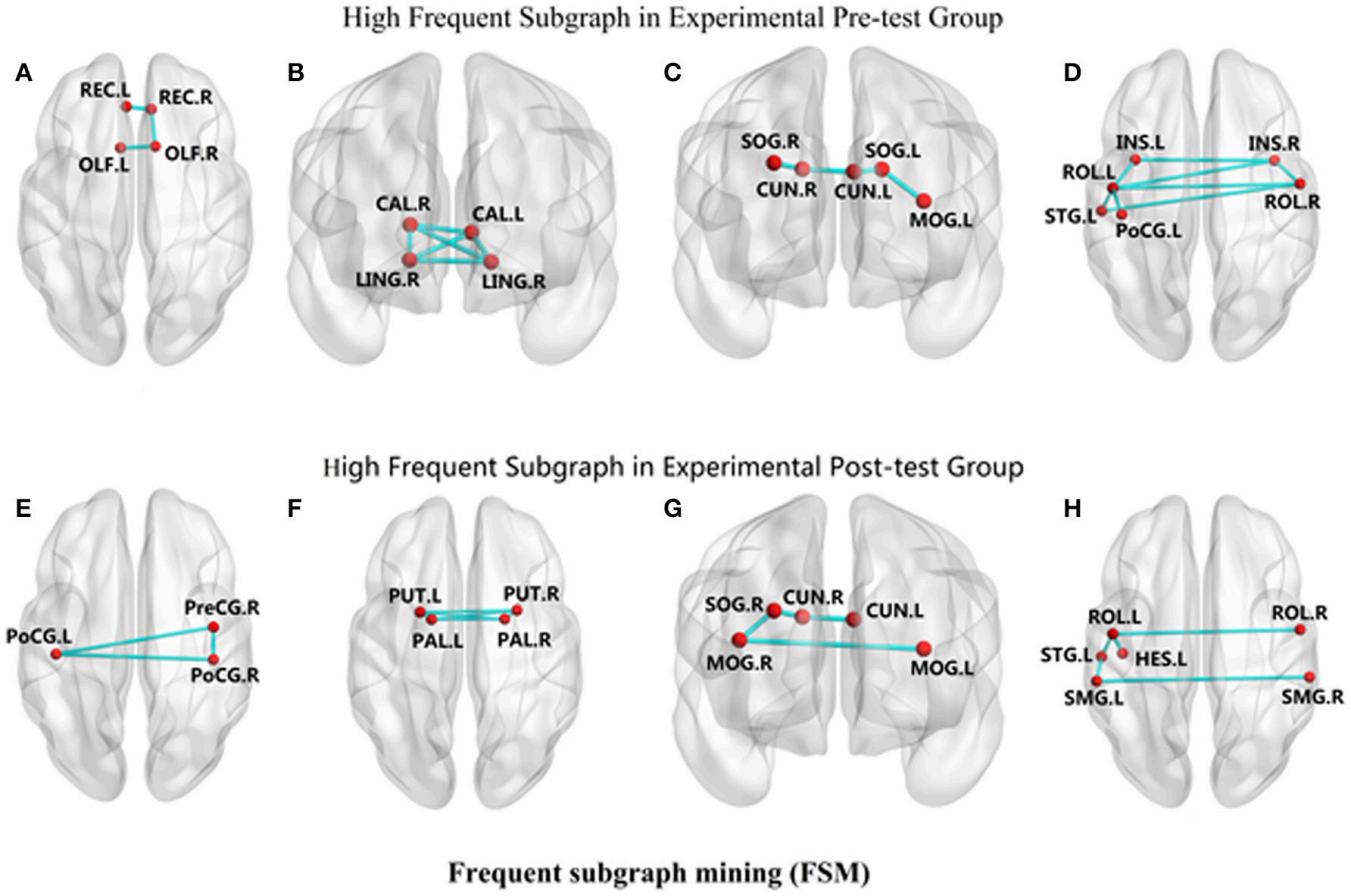
The changes of connectivity patterns in the mined frequent sub-networks between experimental pre-test group and experimental post-test group. The red dots reflect nodes in frequent sub-networks. The links reflect connections in frequent sub-networks. **(A)** REC-OLF, **(B)** CAL-LING, **(C)** CUN-SOG-MOG, **(D)** INS-ROL-PoCG-STG, **(E)** PreCG-PoCG, **(F)** PUT-PAL, **(G)** CUN-SOG-MOG, **(H)** ROL-STG-HES-SMG.

We observed that in CG group, participants had different connectivity patterns in the mined sub-networks between baseline and follow-up. Specifically, after taking CG, new connections frequently appeared for the precentral gyrus-postcentral gyrus (PreCG-PoCG) and putamen-pallidum (PUT-PAL), while some original frequent connections disappeared such as in calcarine-lingual gyrus (CAL-LING) and olfactory-rectus gyrus (OLF-REC). In addition, frequent function connections had changed in some sub-networks. Connectivity pattern changes are shown in Figure 4.

### Correlations between the subtraction of nodal metrics and cognitive function

Correlations between the subtraction of nodal metrics and cognitive function are listed in Table 2. We found that the subtraction (end of intervention—baseline) of nodal metrics correlated with cognitive function changes. For example, the increase in the FIQ score was significantly related to the change of HES.L' nodal efficiency (*p* = 0.020). The increase in the Associate score was marginally related to the change of INS.L' nodal degree (*p* = 0.071) and FFG.R's nodal degree (*p* = 0.073) and nodal efficiency (*p* = 0.083) and the change of OLF.R's nodal efficiency (*p* = 0.067) and betweenness centrality (*p* = 0.097). The increase in the Visual Reproduction score was marginally related to the change of ACG.R' nodal degree (*p* = 0.083) and nodal efficiency (*p* = 0.083). The increase in the Block Design score was also marginally related to the change of ACG.R' nodal degree (*p* = 0.083).

**Table 2:** Correlations between the subtraction of nodal metrics and cognitive function (*n* = 26).

	**Nodal metrics**	**Cognitive function**	**Brain regions**	***r***	**Adjusted *p***	**Effect size**
1	Degree	Associate	INS.L	0.402	0.071^*^	0.878
2		Associate	FFG.R	−0.381	0.073^*^	0.824
3		Visual reproduction	ACG.R	0.350	0.083^*^	0.747
4	Nodal efficiency	Associate	OLF.R	−0.427	0.067^*^	0.944
5		Associate	FFG.R	−0.357	0.083^*^	0.764
6		Block design	ACG.R	0.364	0.083^*^	0.782
7		Visual Reproduction	ACG.R	0.350	0.083^*^	0.747
8		MQ	PAL.R	−0.413	0.071^*^	0.907
9		FIQ	HES.L	0.523	0.020^**^	1.227
10	Betweenness centrality	Associate	OLF.R	−0.333	0.097^*^	0.706
11		Picture completion	CAU.R	−0.385	0.073^*^	0.834

Degree, Nodal Efficiency and Betweenness Centrality are three nodal metrics. FIQ represents the full scale of intelligence quotient and MQ represents the full scale of memory quotient. Picture Completion and Block Design are subscales of WAIS, and Visual Reproduction and Associate are subscales of WMS. Brain regions are some regions in AAL (INS.L, the left insula; FFG.R, the right fusiform gyrus; ACG.R, the right anterior cingulate and paracingulate gyri; OLF.R, the right olfactory cortex; PAL.R, the right pallidum; HES.L, the left heschl gyrus; CAU.R, the right caudate nucleus). r score represents the correlation coefficients between the subtraction of nodal metrics and cognitive function. Adjusted p represents the significance level after correcting for multiple comparisons (

** represents the positive results, and

* *represents the weakly positive results)*.

### Integrative results

**Figure 5:**
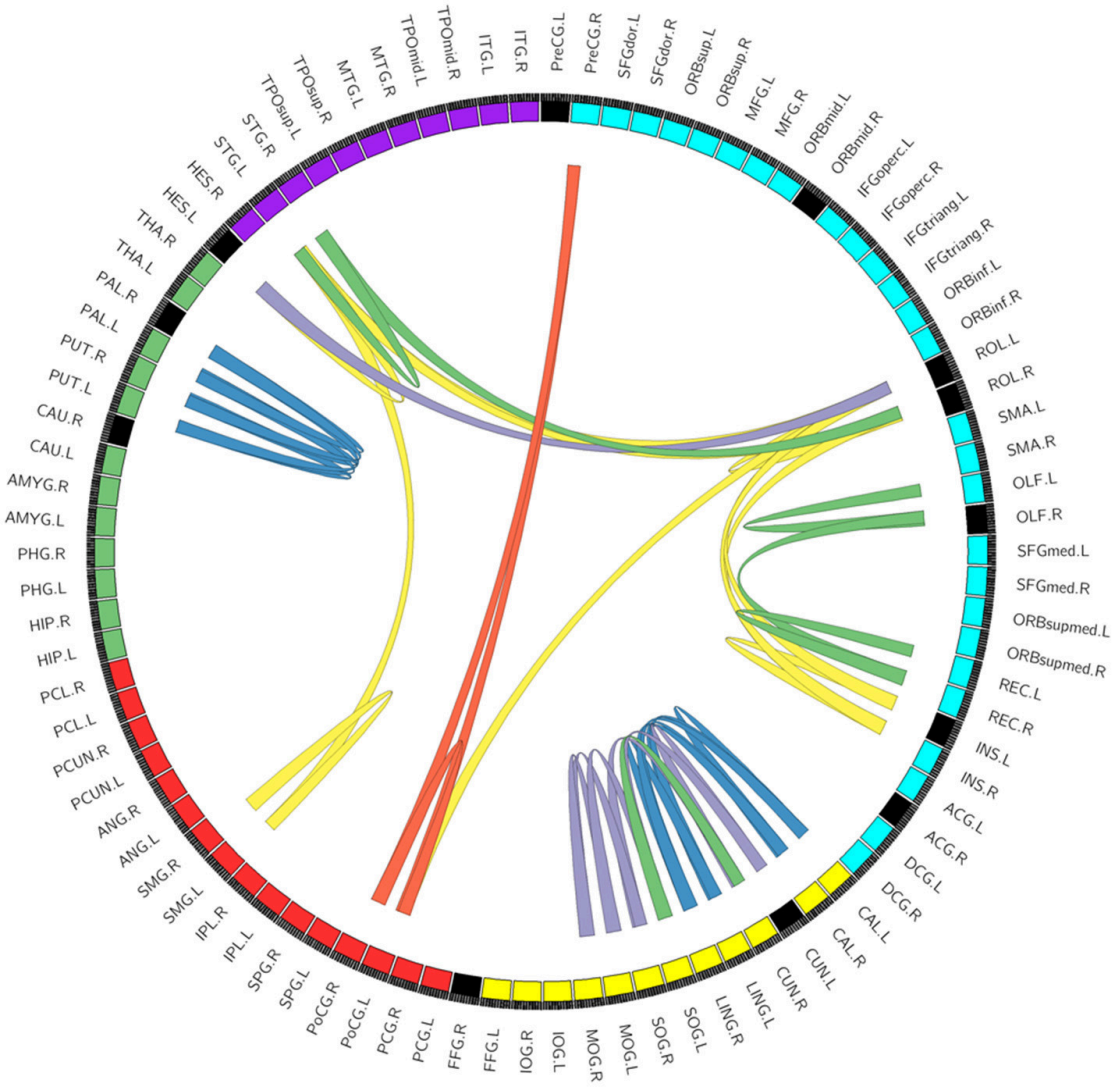
The integrative result of the changes in network topological properties and frequent sub-networks after taking CG. The circle segment reflects the CNM results. Every circle segment reflects a node. Circle segment color reflects module: blue, yellow, red, green, and purple reflects frontal lobe, occipital lobe, parietal lobe, subcortical brain regions, and temporal lobe, respectively. Among those, black circle segments reflects alterant nodes that we found using conventional network metrics (CNM). And the lines in circle reflects the FSM results. We ordered 2–6 edges can constitute a sub-network. Lines color reflects the number of edges in connection pattern in different sub-networks: blue, yellow, purple, green, red reflects 6, 5, 4, 3, and 2 edges, respectively (the repetition links doesn't display). For example, Figure 4A shows a sub-network (three edges) in REC-OLF, which has been shows in the right of image with green lines.

We compared the network topological properties and frequent sub-networks between baseline characteristics and intervention outcomes. The integrative result of changes in network topological properties and frequent sub-networks after taking CG is shown in Figure 5. The circle segment reflects the CNM results, and the lines in circle reflects the FSM findings.

## Discussion

In the present study, we employed a tool set, unitized by WAIS and WMS, to assess cognition changes in sub-healthy participants. Our findings showed that taking CG can improve intelligence and memory, which is similar to previous results reported in mild cognitive impairment patients (Liu et al., 2008). While, without taking CG, there was no change in sub-healthy people's intelligence and memory. Based on these results and our previous work (Xu et al., 2015; Guo et al., 2016), CG can enhance cognition in sub-healthy subjects, especially in intelligence and memory, and this might improve their sub-healthy status.

Based on the tests of cognition, we utilized graph-based network analysis to explore the mechanism of CG. Specifically, we used resting-state fMRI data to construct functional brain network and extract sub-networks. Sorting the results of the study, we found that after taking CG, the nodal metrics of functional brain networks were altered in various frontal, temporal, and occipital cortex regions, as well as several subcortical brain regions. The nodes involved in the FSM results are largely consistent with the CNM findings. We speculate that CG can invoke changes in these regions, including essential components in the executive attention network, the salience network, and the sensory-motor network, which are all involved in cognitive activity. The executive attention network including the bilateral dorsal paracingulate, rolandic operculum, and anterior insula (Vaden et al., 2016) is thought to represent a system that regulates tonic alertness (Sadaghiani and D'Esposito, 2015) and facilitates adaptive control to optimize task performance by monitoring outcomes and initiating behavioral changes (Dosenbach et al., 2006); The salience network, formed by the anterior cingulate cortex and anterior insula, functions to segregate the most relevant internal and external cognitive information to guide behavior (Menon and Uddin, 2010; Palaniyappan and Liddle, 2012). The sensory-motor network including the precentral, postcentral, medial frontal gyri and supplementary motor area (Mantini et al., 2007) is the underlying network for cognition (Meletti et al., 2015). Specially, we found that changes in the ACG, which has an important role in the executive attention and salience networks, was related to improved cognitive function in Visual Reproduction. These findings are consistent with previous studies. For instance, amplitude of low-frequency fluctuation in the cingulate gyri has been positively correlated with visual reproduction (Zhang J. X. et al., 2016). Other researchers have proved that the ACG is a critical structure for social cognitive processing (Rogers et al., 2004; Fujiwara et al., 2007) and memory (Weible et al., 2012; Wartman et al., 2014).

Besides the above regions, there are some positive results in other areas. Typically, the FFG, whose importance in networks has improved after taking CG, was related to intelligence in the Associate test. Similarly, and consistent with our results, regional homogeneity in the FFG.R has been positively correlated with intelligence measured by the WAIS (Wang et al., 2011). In addition, in subcortical brain regions, the functional connection increased in PUT-PAL increased after taking CG, and changes in the PAL were also related to intelligence. Similarly, some researchers have demonstrated that the basal ganglia including the caudate nucleus and pallidum is an important area for a variety of cognitive functions, and focal lesions of this area induce explicit memory deficits (Seger, 2006; Moon et al., 2015). Moreover, the CUN.L (Mochizuki et al., 2014; Li et al., 2016), and MOG.R (Stephan-Otto et al., 2016; Yin et al., 2016) have also been associated with cognition.

However, there are several different regions between the positive results in CNM and FSM, such as the ACG, FFG, and ORBmid. These discrepancies may be due to methodologic differences or study limitations. First, due to the limitation of time and funding, we don't acquire the imaging data of the control group, which should be added in the follow-up study. Second, although participants in the study were sub-healthy, their intelligence and memory levels (FIQ: 103–137; MQ: 100–145) were above average due to their high education. The education background of subjects should be considered in the follow-up studies. Third, cognitive function contains many domains that can be measured in different ways. CG has many components and targets and may likely influence psychological state in addition to cognition. In future investigations, we should explore more concrete psychological problems using targeted approaches. Furthermore, it is important to evaluate participants' status after a 1-year intervention in a longitudinal study. In summary, our findings suggest that CG administration would improve the cognition of sub-healthy individuals by affecting spontaneous brain activity. These results provide a foundation for future studies on the physiological mechanism of CG.

## Ethics statement

All subjects gave written informed consent in accordance with the Declaration of Helsinki. The protocol was approved by the Ethics Committee of the Shanxi Medical University.

## Author contributions

YX and HZ designed and supervised the study. JL, HG, and LG drafted the manuscript. LC, JW, and HL carried out the experimental procedures. JX and JC undertook the statistical analyses and reviewed the literature. KZ participated in data processing.

### Conflict of interest statement

The authors declare that the research was conducted in the absence of any commercial or financial relationships that could be construed as a potential conflict of interest.
