# Supplementary material for: Mechanism of Cerebralcare Granule® for Improving Cognitive Function in Resting-State Brain Functional Networks of Sub-healthy Subjects
Source: Front Neurosci. 2017 Jul 14;11:410. doi: 10.3389/fnins.2017.00410 (PMC5509764; doi:10.3389/fnins.2017.00410)
Supplement: Supplementary file 1 [file Presentation1.PDF]

Supplementary Material

Exploring the Mechanism of Resting-state Brain Functional Networks for Cerebralcare Granule® Improving Cognitive Function in Sub-healthy People

Jing Li[1†], Hao Guo[2†], Ling Ge[1,3†], Long Cheng[4], Junjie Wang[1], Hong Li[1], Kerang Zhang[4], Jie Xiang[2], Junjie Chen[2], Hui Zhang[5*†] and Yong Xu[4,6*†]

*** Correspondence:**
Yong Xu: xuyongsmu@vip.163.com
Hui Zhang: zhanghui_mr@163.com

1 Mathematical Definitions of Nodal Metrics

Unless otherwise mentioned, all formulas introduced below are based on $G = (N,K)$, a network G with N nodes and K edges. Three nodal measures were examined in the current study: degree(k_i), betweenness centrality(b_i) and nodal efficiency(e_i).

Degree

Formally, the degree of node i is defined as:

$$k_i = \sum_{j \in G} a_{ij} \tag{1}$$

where a_{ij} is the element (i,j) in the network. Degree is a simple measurement of connectivity of a node with the rest of nodes in a network.

Nodal efficiency

The nodal efficiency of node $i$is computed as (Achard & Bullmore, 2007):

$$e_i = \frac{1}{N-1} \sum_{j \neq i \in G} d_{ij} \tag{2}$$

where d_{ij} is the shortest path length between node i and node j in G. The shortest path length is the minimum number of edges for the network among all possible paths from one node to another in G. Nodal efficiency measures the ability of information propagation between a given node i with the rest of nodes in a network.

Betweenness Centrality

The betweenness centrality of node is measured as (Freeman, 1977):

$$b_i = \sum_{m \neq i \neq n \in G} \frac{\sigma_{mn}(i)}{\sigma_{mn}} \tag{3}$$

where σ_{mn} is the total number of shortest paths (paths with the shortest path length) from node m to node n, and $\sigma_{mn}(i)$ is the number of shortest paths from node m to node n that pass through the node i. Betweenness centrality of a node captures the influence

of the node over information flow between all the other nodes in the network.

2 gSpan Algorithm

The gSpan algorithm used in this research, the main advantages of the algorithm include: (1) a kind of depth first enumeration framework is designed for efficient traversal graph pattern search space; (2) the anti monotonicity properties based on support, using the branch and bound algorithm to prune the search space pattern, to improve the efficiency of mining; (3) when calculating the degree of support, some optimization strategies are designed. In some conditions, the mining efficiency can be improved obviously by using the linked list method. We give some definitions in graph mining (Huan et al., 2003) and gSpan algorithm description (Yan et al., 2002) below.

Labeled Undirected Graph

Let G = (V, E, L, l) be a labeled undirected graph, where V is a set of nodes and $E \subseteq V \times V$ ·V is a set of edges. e = {u,v} indicates an edge between the nodes u and v. L is a set of labels, and l is a mapping function that assigns labels to vertices in V and edges in E.

Subgraph

For two labeled undirected graphs, $G_S = (V_S, E_S, L_S, l_S)$ and G = (V, E, L, l), Gs is a subgraph of G if $V_S \subseteq V, E_S \subseteq E, L_S \subseteq L, l_s \subseteq l$,.

Graph Isomorphism

A graph $G_1 = (V_1, E_1, L_{V1}, L_{E1}, l_1)$ is isomorphic to another graph $G_2 = (V_2, E_2, L_{V2}, L_{E2}, l_2)$, if and only a bijection $f: V_1 \rightarrow V_2$ exists such that

(i) $\forall u \in V_1, l_1(u) = l_2(f(u))$,

(ii) $\forall (u, v) \in E_1 \Leftrightarrow (f(u), f(v)) \in E_2$,

(iii) $\forall (u, v) \in E_1, l_1(u, v) = l_2(f(u), f(v))$,

The bijection f is an isomorphism between G1 and G2.

Subgraph Frequency Ratio

Given a set of graphs, G, the frequency ratio of a subgraph g_s, is defined as

$$fq(g_s|G) = \frac{|g_s \text{ is a subgrapg of } g, g \in G|}{G}$$

Frequent Subgraph

Given a set of graphs, G and a support parameter s, a subgraph $g_s$is a frequent subgraph if and only g_s exists in at least $S \cdot |G|$ of the input graph set.

gSpan Algorithm

The idea of the gSpan algorithm: 1) gSpan first constructs a new lexicographic order among graphs, and maps each graph into a unique minimum DFS code as its canonical label.2) then, based on the lexicographic order, gSpan utilizes the DFS strategy to mine frequent connected subgraph patterns efficiently. 3) Finally, all

subgraphs with nonminimal DFS code are pruned to avoid redundant candidate generations . Its pseudo code is as follows:

```
Input: G
Output: frequent subgraph S
1   Sort the labels in G according to the frequency;
2   Remove infrequent vertices and edges;
3   Relabel the remaining vertices and edges;
4   S1← all frequent 1-edge graphs in G;
5   Sort S1 in DFS lexicographic order;
6   S ← S1;
7   for each edge e∈ S1 do
8   Initialize s with e, set s.G by graphs which contain e;
9   if s ≠min(s) then
10    return;
11    end
12    S ←S U s;
13    Enumerate s in each graph in G and count its children;
14    for each c, c is s'child do
15    if support(c)≥ minSup then
16    s←c;
17    end
18    Go to step 9;
19    G←G -e;
20    if |G| ≤ minSup then
21    Break;
22    end
23    end
24    end
```

Algorithm 1: frequent subgraph mining algorithm gSpan

3 Principal gender characteristics of participants

gender	test	FIQ			MQ		
		means ±standard deviation	*t*	*p*	means ±standard deviation	*t*	*p*
Female	One-Sample Kolmogorov-Smirnov Test	120.29 ±9.191	-	0.922	121.92 ±8.934	-	0.726
Male		123.00 ±8.741	-	0.834	116.67 ±11.827	-	0.902
Female	Levene's Test for Equality of Variances	120.29 ±9.191	-	0.847	121.92 ±8.934	-	0.366
Male		123.00 ±8.741			116.67 ±11.827		
Female	Independent Samples Test	120.29 ±9.191	0.651	0.520	121.92 ±8.934	-1.209	0.237
Male		123.00 ±8.741			116.67 ±11.827		

One-Sample Kolmogorov-Smirnov Test showed that the distribution of the female’s and male’s FIQ and MQ is Normal, and Levene’s Test for Equality of Variances showed that difference genders had equal variances in FIQ and MQ. Then, consider the normal distribution and homogeneity of variance, we used Independent Samples Test in testing the differences between female and male, and the results showed that different genders had no influence on FIQ and MQ.

References

Achard, S., Bullmore, E. (2007). Efficiency and cost of economical brain functional network. *PLOS Computation Biology* 3, e17.

Freeman, L.C. (1977). A set of measures of centrality Based on Betweenness. *Sociometry* 40, 35-41.

Huan, J., Wang, W., Prins, J. (2003). Efficient mining of frequent subgraph in the presence of isomorphism. *IEEE International Conference on Data Mining,* 549-552.

Yan, X.F., Han, J.W. (2002). gSapn: Graph-based substructure pattern mining. *IEEE International Conference on Data Mining*, 721-724.
